# Parental burnout and sleep problems in Iranian mothers of primary school‐aged children: Exploring the mediation effect of emotional schemas

**DOI:** 10.1002/brb3.2688

**Published:** 2022-07-18

**Authors:** Bessat Kalantar Hormozi, Zohreh Khosravi, Narges Sabzi

**Affiliations:** ^1^ Faculty of Education and Psychology, Department of Psychology Alzahra University Tehran Iran; ^2^ Faculty of Education and Psychology, Department of Psychology Alzahra University Tehran Iran

**Keywords:** emotional schemas, hypersomnia, insomnia, parental burnout, sleep problems, structural equation modeling

## Abstract

**Objectives:**

Sleep is crucial for mental well‐being. Evidence suggests sleep problems in mothers can result from parental burnout. The possible mediators that link parental burnout to sleep problems have not been investigated. This study seeks to explore the mediational role of emotional schemas as psychological constructs, which relate parental burnout to sleep problems in mothers of school‐aged children.

**Method:**

A total of 224 mothers participated voluntarily in this cross‐sectional study. Data were collected online. The participants completed Parental Burnout Assessment (PBA) scale, Mini Sleep Questionnaire‐Persian Version (MSQ‐P), and Leahy Emotional Schema Scale (LESS II). Structural equation modeling (SEM) was performed using the bootstrap method to assess the mediation model.

**Results:**

The findings of this research indicate a positive correlation exists between parental burnout, emotional schemas, and insomnia/hypersomnia. The mediation analysis confirmed parental burnout and insomnia/ hypersomnia are related indirectly through emotional schemas.

**Conclusion:**

Implications of the findings is that when parental burnout is present, the psychological treatment of sleep problems may benefit from targeting emotional schemas. However, further research is needed to determine whether similar mediational effects are replicated.

## Graphical abstract

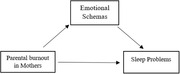

## 1 INTRODUCTION

Sleep is essential for maintaining psychological and physical health. Poor sleep can interact with psychological and physical disorders and impose several health consequences (Medic et al., 2017). Studies indicate insufficient sleep is associated with diabetes (Cappuccio et al., 2009), cerebral–vascular disorders (Hoevenaar‐Blom et al., 2011), cardiometabolic outcomes (Altman et al., 2012), and increased mortality (Kripke et al., 2002). Numerous studies have examined the negative impacts of COVID‐19 on sleep (Alimoradi et al., 2021; Dellagiulia et al., 2020; Jahrami et al., 2021; Lin et al., 2021; Sher, 2020; Sinha et al., 2020). Also, studies that investigated sleep patterns in parents during the pandemic report modifications in sleep patterns and quality such as increased duration, more dreams and later rising (Altena et al., 2020; Curtis et al., 2021).

In general, low socioeconomic status, genetic factors, age‐related cognitive and physiological changes, personality factors (such as perfectionism, reduced self‐esteem, introversion and internalization), working conditions, and major life events that increase stress levels and being female are considered to be predictors of poor sleep quality (Stuck et al., 2021). Additionally, studies show during the current pandemic mothers are anticipated to have more sleep problems than fathers (Wearick‐Silva et al., 2021).

During the global COVID‐19 pandemic and the consequent lockdowns, mothers, as primary caregivers of children have experienced changes related to their roles. The closure of schools as a measure to maintain social distancing during the COVID‐19 and the burden of homeschooling has impacted mothers negatively (Calarco et al., 2020; Cluver et al., 2020; Petts et al., 2021; Schmidt et al., 2021; Thorell et al., 2021). When mothers’ well‐being is threatened, there will be complications for both the mother and the child (Nomaguchi & Milkie, 2020), which makes it necessary to attend to mental health issues and their correlates in mothers.

Recent evidence suggests sleep problems in parents can be evaluated as the consequence of parental burnout (Mikolajczak et al., 2018). Parental burnout is characterized by feeling exhausted and unhappy because of the parental role, feeling different from previous parental self, and emotional distancing from offspring (Mikolajczak et al., 2018). Roskam and Mikolajczak (2020) studied the average level of parental burnout among fathers and mothers and reported higher levels of burnout in mothers. According to another research, which investigated parental burnout in Iranian parents during COVID‐19 quarantine in Iran, mothers report significantly higher parental burnout than fathers (Mousavi, 2020).

Possible psychological mechanisms, which may help explain how parental burnout is linked to sleep, are not yet identified. The diathesis‐stress model for psychological disorders suggests insomnia occurs in relation to both predisposing and precipitating factors and that the chronic form of the disorder is maintained by maladaptive coping behaviors (perpetuating factors). Psychological factors play a major role in the etiology and maintenance of insomnia (Stuck et al., 2021).

Among psychological treatments available for insomnia, cognitive‐behavioral therapy (CBT‐I) has been validated and is considered to be the main treatment for insomnia. CBT‐I is even preferred to pharmaceutical medications for insomnia (Stuck et al., 2021; Taylor et al., 2015). Similar to other psychological treatments, cognitive‐behavioral therapy for insomnia has been evolving in the recent years. Today, third‐wave therapies, which focus on values, skills instead of symptoms and emotional processes, are being tested as insomnia treatments (Kim et al., 2021; Perini et al., 2021; Salari et al., 2020; Taylor et al., 2015).

“Emotional schemas” or beliefs about emotion are ways of understanding and regulating emotions and are the result of cognitive assessment of emotion. These schemas are individualized and are shaped by person‐specific experiences such as parenting and emotion socialization, family environment, cultural influences, and the individual's history of trauma (Edwards, 2019; Leahy, 2015). The 14 dimensions of emotional schemas are: validation (believing others can understand, tolerate and show empathy for one's feelings), rumination (repeatedly refocusing on negative experience, thoughts, and emotions), comprehensibility (feeling confused and helpless about emotions), simplistic views of emotion (having difficulty with mixed feelings), values (believing emotions are natural consequences of the values that direct one's life), control (believing that one needs to do almost anything to control emotions), numbness (feeling detached from reality due to emotional avoidance and insufficient emotional processing), rationality (believing emotions should be eliminated or controlled in order to stay rational in everything), duration (tendency to see negative emotions as permanent or, personality “traits”), consensus (tendency to believe one's emotions are abnormal), guilt (criticizing oneself for having certain emotions and believing one should hide emotions), acceptance (allowing oneself to have certain feelings), expression (believing one can communicate one's feeling to others openly), and blame (believing others are the cause of one's negative feelings). Previous research reveals certain emotional schemas have associations with psychopathologies and can also elicit dysfunctional emotion regulation strategies such as rumination, avoidance, or worry (Edwards & Wupperman, 2019; Silberstein et al., 2012). Research results show depression is related to emotional schemas of guilt, rumination, and expectation of longer duration (Leahy et al., 2012). Mood disorders have shown to be related to simplistic view of emotions, rumination, numbness, values, rationality, and control emotional schemas (Batmaz et al., 2014). Emotional schemas have also been studied as mediators. Edwards et al. (2016) suggest emotional schemas mediate the association between early childhood abuse and later alexithymia. The link between emotional schemas and sleep is traceable in the available literature. Rumination is shown to be associated with sleep disturbances (Clancy et al., 2020; Pillai & Drake, 2015). Available studies indicate that dysfunctional emotion regulation strategies can lead to sleep disturbances (Gruber et al., 2008; Guastella & Moulds, 2007). Tsypes et al. (2013) identified difficulties with emotion regulation as a mediator of the relationship between generalized anxiety disorder diagnosis and problems with sleep.

**FIGURE 1:**
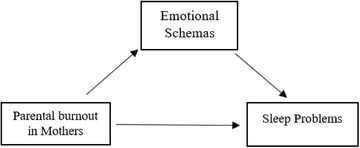
The conceptualized model of the study

To our knowledge, there is no research available regarding sleep quality in Iranian mothers’ during COVID‐19 pandemic, which investigates the relationships among parental burnout, emotional schemas, and sleep problems. Exploring these relationships may deliver insight for clinicians who use cognitive behavioral therapies for sleep disorders. In the present study, we will specifically investigate whether (a) parental burnout is positively correlated to sleep problems, (b) parental burnout is positively correlated to maladaptive emotional schemas, (c) maladaptive emotional schemas are positively correlated to sleep problems, (d) emotional schemas mediate the relationship between parental burnout and sleep problems. Figure 1 demonstrates the conceptualized model for this study.

## 2 METHODS

### 2.1 Participants and procedures

In this cross‐sectional study, 224 mothers participated voluntarily from the general population. Data were collected through completion of self‐report questionnaires on Google form, which was shared on Instagram, Telegram, and whatsApp groups, and were also announced by colleagues. An information note describing the objectives of the study, researchers’ contact details, and inclusion/exclusion criteria was included in the first page of the form, and participants were asked to consent to taking part in the research before gaining access to the questionnaires. Inclusion criteria included (1) being a mother with primary school‐aged children and (2) willingness to complete the research questionnaires voluntarily. Exclusion criteria included (1) having been previously diagnosed with psychological disorders or any physical conditions (so if necessary, they could be excluded by researchers during screening), (2) taking medication that impacts sleep, and (2) having a child or family member with special needs/ disabilities.

The participants were assured of the confidentiality and anonymity of the provided information. Approximately 20 min was needed for completing the questionnaires and the design of the survey in the Google form was set not to allow for missing responses.

This study was approved by Alzahra University ethics committee (IR.ALZAHRA.REC.1400.071).

### 2.2 Measures

#### 2.2.1 Demographic questionnaire

Participants’ age, marital status, educational level, employment status, and number of children were recorded by the Demographic questionnaire.

#### 2.2.2 Parental burnout

Parental burnout is evaluated by self‐report Parental Burnout Assessment (PBA) scale, which includes 23 items with a 7‐point Likert scale for responses that covers *never* (0 points) to *every day* (6 points). This questionnaire was initially constructed by Roskam et al. (2018) and examines four subscales: exhaustion of parental role, contrast with the parental self in the past, feeling of being fed up, and emotional distancing. The psychometric characteristics of this scale were validated in Iranian population by Mousavi et al. (2020) who reported Cronbach's alpha values from .60 to .93 for different subscales.

#### 2.2.3 Sleep disorders

Mini Sleep Questionnaire‐Persian Version (MSQ‐P) was used to evaluate sleep problems among participants. The mini Sleep questionnaire (MSQ) was originally developed by Zomer et al. (1985) and later underwent changes. The current MSQ contains 10 items and each item uses a 7‐point Likert scale (1, never; 4, sometimes; 7, always). Falavigna et al. (2011) reported Cronbach's alpha value of .77 for this questionnaire, which demonstrated good internal consistency. Reliability and validity of MSQ‐Persian version were confirmed by Hosseini et al. (2020) who reported Cronbach's alpha value of .75 and test–retest correlation coefficient of .91.

#### 2.2.4 Emotional schemas

Farsi version of Leahy Emotional Schema Scale II (LESS II) was used to explore emotional schemas in the participants. This 28‐item questionnaire measures the 14 dimensions of emotional schemas and evaluates one's assessment and responses to emotional experience (Suh et al., 2019). These 14 dimensions include invalidation, rumination, incomprehensibility, guilt, simplistic view of emotion, not being valued, numbness, loss of control, rationality, duration, low consensus, nonacceptance of feelings, low expression, and blame. LESS II has shown an acceptable Cronbach's alpha value (.76) and a significant concurrent validity (*r* = .73, *p* < .05) with the Beliefs about Emotions Scale (BAES) (Batmaz & Ozdel, 2015). The Farsi version of LESS II has Cronbach's alpha value of .74 and test–retest coefficient of .71 (Salemi‐Langroudi et al., 2021).

#### 2.2.5 Statistical analysis

The collected data were analyzed using SPSS 26. For exploring the paths in the mediation model, structural equation modeling (SEM) was performed using the bootstrap method in AMOS 24.

In the proposed model for this research, sleep problems were the endogenous variable, parental burnout was considered to be the exogenous variable, and emotional schemas was the mediating variable.

## 3 RESULTS

### 3.1 Demographic characteristics of the sample

The sample consisted of 224 participants (*n* = 224). The age range was 25–51 with mean age 36.8 (*SD* = 5.0). Regarding marital status, 209 (93.3%) were married, 12 (5.4%) were divorced, and 3 (1.3%) were widowed. %65.2 (*n* = 146) were homemakers and %34.8 (*n* = 78) were employed. %17.4 (*n* = 39) had graduate degrees, %49.1 (*n* = 110) had bachelor's degrees, and %33.5 (*n* = 75) did not attend university. %31.3 (*n* = 70) had only one child, % 52.2 (*n* = 117) had two children, and % 16.4 (*n* = 37) reported having three or more children.

### 3.2 Descriptive statistics

**FIGURE 2:**
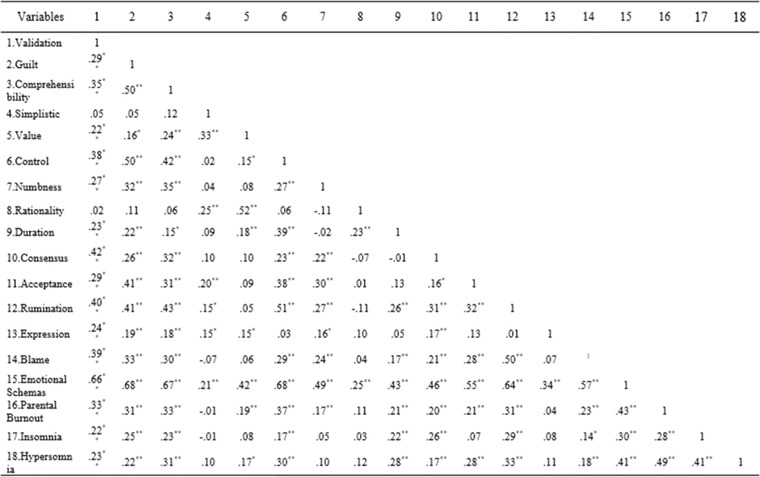
Correlation matrix

The skewness and kurtosis values were between −2 and +2 so data distribution was normal. Multivariate normality was checked using Mardia's coefficient and Mahalanobis distance and outliers were corrected according to the obtained values. Table 1 reports descriptive statistics for research variables. Correlations were calculated using Pearson's coefficients. Figure 2 depicts the correlation matrix.

**TABLE 1:** Descriptive analysis

Kurtosis	Skewness	SD	Mean	Variables
−0.89	0.27	2.82	6.63	1. Validation
−0.78	0.32	2.64	5.79	2. Guilt
−0.35	0.67	2.42	4.59	3. Comprehensibility
2.24	1.18	1.68	3.75	4. Simplistic
0.77	0.99	1.98	4.22	5. Value
−1.14	0.16	2.99	6.08	6. Control
−0.32	0.56	2.62	5.29	7. Numbness
1.07	1.14	2.28	4.39	8. Rationality
−0.53	0.24	2.40	5.94	9. Duration
−0.37	−0.09	2.35	7.10	10. Consensus
−0.44	−0.14	2.06	5.72	11. Acceptance
−0.87	−0.14	2.89	7.30	12. Rumination
0.29	0.58	2.14	5.67	13. Expression
−0.52	−0.43	2.76	7.85	14. Blame
−0.57	0.05	17.79	80.34	15. Emotional schemas
1.38	1.53	35.29	30.85	16. Parental burnout
−0.18	0.30	3.99	12.70	17. Insomnia
−0.27	0.50	6.07	17.18	18. Hypersomnia

Pearson's correlations show significant positive correlations between all latent variables (*p* < .05).

The correlation coefficient for insomnia and parental burnout was .28, and for insomnia and emotional schemas .30. Among emotional schemas, insomnia had the highest correlation with rumination (.29). The discriminant validity was assessed using Fornell and Larcker's (1981) criteria. Factor loadings that were at least .30 were kept in the model. Cronbach's alpha values for PBA questionnaire, LESS II, insomnia, and hypersomnia were respectively .92, .82, .73, and .76. Composite reliability was as follows: parental burnout .94, emotional schemas .83, insomnia .79, and hypersomnia .80.

### 3.3 The measurement model

**FIGURE 3:**
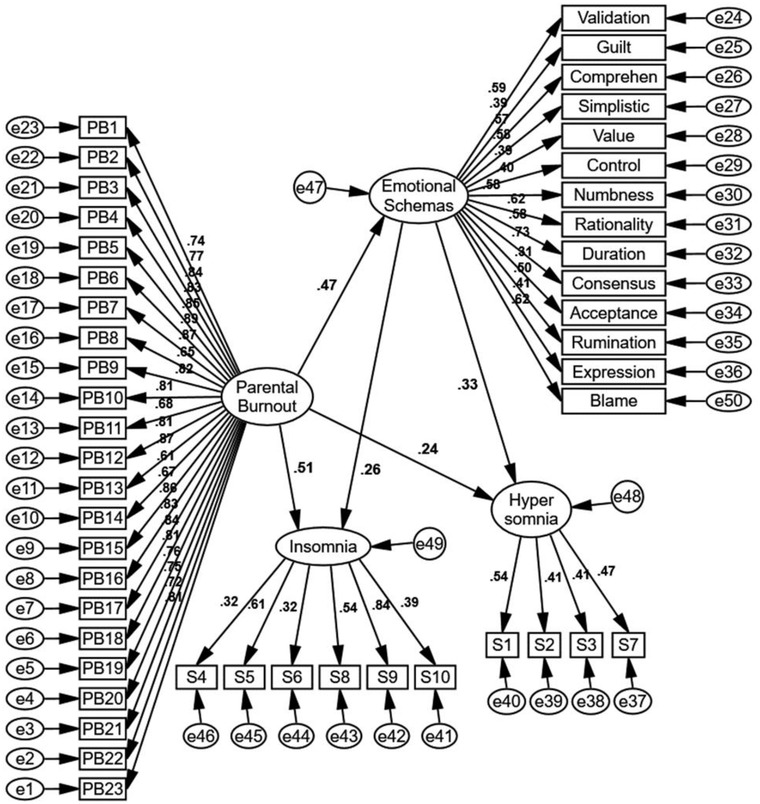
Measurement model in the standard mode

**FIGURE 4:**
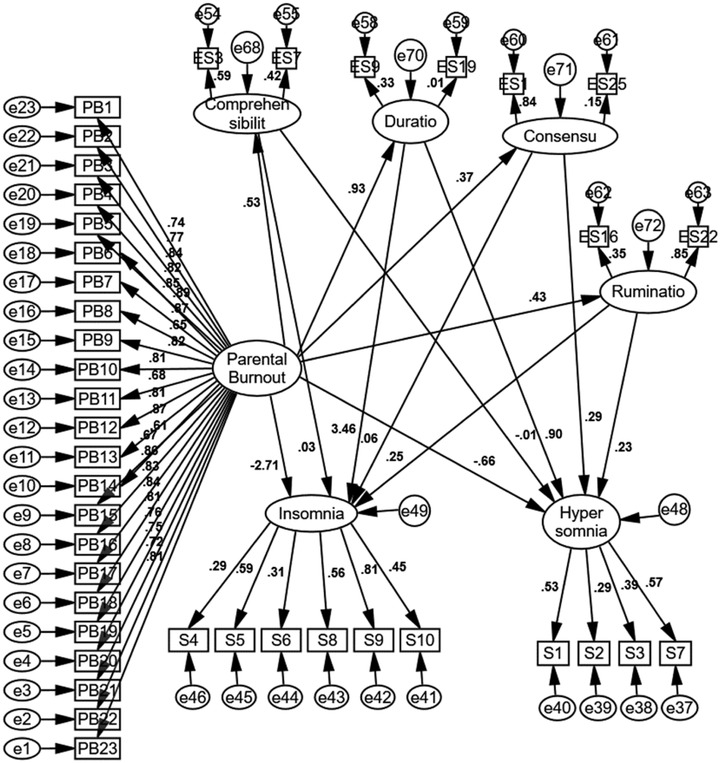
The complementary model

Figure 3 presents the measurement model drawn in AMOS software in standard mode. As can be seen, the strongest correlations were between parental burnout and emotional schemas, and parental burnout and insomnia. Fit indices of the model are reported in Table 2. Overall, fit indices indicate the model is an acceptable fit. Coefficient of determination was .46 for insomnia and .26 for hypersomnia, which is a acceptable considering there are two variables (parental burnout and emotional schemas) affecting insomnia and hypersomnia. Results demonstrate that all direct paths are confirmed (*p* < .05). The mediation model was assessed using the bootstrap method in AMOS. Table 3 reports the regression weights for the direct paths. Table 4 that presents the bootstrap results indicates that the conceptualized model is confirmed and emotional schemas have a mediational role in the relationship between parental burnout and sleep problems (hypersomnia and insomnia) (*p* < .05). To investigate whether certain emotional schemas mediate the association between parental burnout and sleep issues, a complementary model was run (Figure 4). Although when considered as a single variable emotional schemas act a mediator, the results (which are available in Appendix) show when single schemas are investigated as the mediator, many paths are not significant. This may be due to low number of items for each emotional schema.

**TABLE 2:** Fit indices of the model

Fit indices	AGFI	PGFI	IFI	NFI	CFI	GFI	RMSEA	Chi square/df
**Criteria**	.70<	.70<	.90<	.90<	.90<	.90<	.08 >	Between 1 and 5
**Results**	.71	.73	.88	.93	.92	.93	.078	2.78

**TABLE 3:** Direct paths regression weights

Direct paths	Standardized coefficent	Unstandardized coefficent	SE	*t*	*p*
Parental Burnout → Emotional Schemas	.490	.30	0.05	5.70	<.001
Parental Burnout → Insomnia	.510	.23	0.05	4.41	<.001
Parental Burnout → Hypersomnia	.240	.11	0.04	2.20	.028
Emotional Schemas → Insomnia	.260	.18	0.06	2.86	.004
Emotional Schemas → Hypersomnia	.33	.24	0.08	2.67	.007

**TABLE 4:** Bootstrap results for mediation model

Indirect paths	Indirect effect	SE	*t*	*p*
Parental Burnout → Emotional Schemas → Insomnia	0.122	0.046	2.65	.008
Parental Burnout → Emotional Schemas → Hypersomnia	0.155	0.056	2.77	.006

## 4 DISCUSSION

The purpose of this study was to assess the relationship between parental burnout, emotional schemas, and sleep problems (insomnia and hypersomnia) and also examine the mediational role of emotional schemas in this relationship. Results demonstrated that parental burnout is positively associated with both insomnia and hypersomnia. This finding is consistent with the results from a study by Mikolajczak et al. (2018), which revealed parental burnout's effect on sleep problems is statistically similar job burnout's effect on sleep. Earlier research on burnout and sleep disturbances explains this relationship by pointing to the subjective issues such as emotional exhaustion caused by burnout (Harvey, 2002; Harvey et al., 2008). These subjective issues are considered in this study as emotional schemas, which are investigated in the last hypothesis.

Results revealed a positive association between parental burnout in mothers and emotional schemas, meaning emotional exhaustion and further feelings of detachment caused by burnout are related to higher scores on negative emotional schemas dimensions. This result is aligned with the emotional schema model (Leahy, 2019) that proposes that when one is experiencing emotional hardship, one's beliefs about emotion (emotional schemas) determine how one copes with the circumstances; therefore, higher emotional distress is expected to be accompanied by higher scores in negative emotional schemas.

The results also indicate that emotional schemas are positively related to sleep problems in mothers. This is in agreement with previous studies that highlight the influence of subjective processes such as emotional dysregulation (Vandekerckhove & Wang, 2017) and also metacognitive processes (Palagini et al., 2013; Palagini et al., 2017; Sella et al., 2018) on sleep quality and sleep disorders. This finding could be explained by the diathesis‐stress model. Based on this model, we can consider emotional schemas as potential perpetuating factors that contribute to the maintenance of sleep issues.

The mediation model investigated in this study confirmed the initial hypothesis, which predicted emotional schemas can indirectly relate parental burnout to sleep problems. This finding is consistent with a previous research by Palagini et al. (2017), which suggest subjective psychological processes are involved in the association between burnout and sleep problems.

Because of the negative impact of sleep problems on mental and physical well‐being, it is important to attend contributing psychological factors that can be managed through psychotherapy. Emotional schemas, as transdiagnostic psychological constructs, can be effectively embedded in many forms of therapy. Therefore, the findings of the current research have important implications for interventions and the prevention of sleep issues related to burnout. Additionally, up until now, many studies in the field of parental burnout were designed to evaluate the antecedents of parental burnout. The current study focused on the consequences of parental burnout. Moreover, many studies that concentrate on sleep problems mainly study insomnia. The researchers of the current study included both insomnia and hypersomnia in the study model. Finally, there are few studies available on parental burnout in Iranian mothers; thus the current study contributes to the available literature in this field.

### 4.1 Limitations and implications for further research

This study has a cross‐sectional design and although the findings can be beneficial for later research, to further investigate casual relationships, a longitudinal study is needed. The study was performed among mothers because in the studied population, mothers are assumed to be the primary caregivers and are probably more involved in homeschooling. This may not apply in cultures where fathers may take on more duties related to children and so therefore any generalization should be done cautiously. The data were collected through online self‐report surveys, which means subjective biases and misinterpretations of the questions may have impacted the participants’ responses. When investigating parental burnout, we concentrated on parental burnout total score and did not study its dimensions separately that can be considered in future research. Finally, although the sample size of this research was sufficient for structural equation modeling, it is suggested that it is replicated with a larger sample size in the future.

## 5 CONCLUSION

In conclusion, this study showed emotional schemas can indirectly relate parental burnout and sleep problems in Iranian mothers who are under the pressure of homeschooling during the COVID‐19 pandemic. This finding shed light on the role of emotional schemas in sleep problems. Additionally, it may add to the available research regarding parental burnout, which is a rather new construct.

## CONFLICT OF INTEREST

The authors declare that they have no competing interests.

6

### PEER REVIEW

The peer review history for this article is available at: https://publons.com/publon/10.1002/brb3.2688.

## FUNDING

This research did not receive any funding.

## Data Availability

The data set is available from the corresponding author upon reasonable request.

## APPENDIX A STANDARDIZED REGRESSION WEIGHTS FOR THE SECOND MODEL

### A.1

**Table:**

			Estimate
Comprehen_sibilit	←‐	Parental_Burnout	0.534
Duratio	←‐	Parental_Burnout	0.931
Consensu	←‐	Parental_Burnout	0.370
Ruminatio	←‐	Parental_Burnout	0.427
Hyper_somnia	←‐	Parental_Burnout	–0.657
Insomnia	←‐	Parental_Burnout	−2.714
Hyper_somnia	←‐	Ruminatio	0.230
Hyper_somnia	←‐	Consensu	0.289
Hyper_somnia	←‐	Duratio	0.898
Hyper_somnia	←‐	Comprehen_sibilit	–0.010
Insomnia	←‐	Ruminatio	0.246
Insomnia	←‐	Consensu	0.058
Insomnia	←‐	Duratio	3.457
Insomnia	←‐	Comprehen_sibilit	0.028
PB23	←‐	Parental_Burnout	0.810
PB22	←‐	Parental_Burnout	0.723
PB21	←‐	Parental_Burnout	0.745
PB20	←‐	Parental_Burnout	0.763
PB19	←‐	Parental_Burnout	0.806
PB18	←‐	Parental_Burnout	0.840
PB17	←‐	Parental_Burnout	0.827
PB16	←‐	Parental_Burnout	0.860
PB15	←‐	Parental_Burnout	0.670
PB14	←‐	Parental_Burnout	0.614
PB13	←‐	Parental_Burnout	0.871
PB12	←‐	Parental_Burnout	0.811
PB11	←‐	Parental_Burnout	0.683
PB10	←‐	Parental_Burnout	0.807
PB9	←‐	Parental_Burnout	0.818
PB8	←‐	Parental_Burnout	0.651
PB7	←‐	Parental_Burnout	0.874
PB6	←‐	Parental_Burnout	0.887
PB5	←‐	Parental_Burnout	0.851
PB4	←‐	Parental_Burnout	0.824
PB3	←‐	Parental_Burnout	0.845
PB2	←‐	Parental_Burnout	0.772
PB1	←‐	Parental_Burnout	0.737
S7	←‐	Hyper_somnia	0.568
S3	←‐	Hyper_somnia	0.395
S2	←‐	Hyper_somnia	0.286
S1	←‐	Hyper_somnia	0.535
S10	←‐	Insomnia	0.449
S9	←‐	Insomnia	0.808
S8	←‐	Insomnia	0.559
S6	←‐	Insomnia	0.310
S5	←‐	Insomnia	0.592
S4	←‐	Insomnia	0.293
ES3	←‐	Comprehen_sibilit	0.586
ES7	←‐	Comprehen_sibilit	0.422
ES9	←‐	Duratio	0.327
ES19	←‐	Duratio	0.011
ES1	←‐	Consensu	0.839
ES25	←‐	Consensu	0.146
ES16	←‐	Ruminatio	0.350
ES22	←‐	Ruminatio	0.852

### REGRESSION WEIGHTS FOR THE SECOND MODEL

**Table:**

			Estimate	SE	CR	*p*	Label
Comprehen_sibilit	←‐	Parental_Burnout	0.340	0.072	4.732	***	
Duratio	←‐	Parental_Burnout	0.338	0.073	4.607	***	
Consensu	←‐	Parental_Burnout	0.334	0.071	4.699	***	
Ruminatio	←‐	Parental_Burnout	0.179	0.072	2.475	.013	
Hyper_somnia	←‐	Parental_Burnout	–0.354	0.238	−1.488	.137	
Insomnia	←‐	Parental_Burnout	−1.369	3.400	–0.402	.687	
Hyper_somnia	←‐	Ruminatio	0.295	0.164	1.804	.071	
Hyper_somnia	←‐	Consensu	0.173	0.218	0.793	.428	
Hyper_somnia	←‐	Duratio	1.332	0.598	2.227	.026	
Hyper_somnia	←‐	Comprehen_sibilit	–0.009	0.146	–0.060	.952	
Insomnia	←‐	Ruminatio	0.296	0.125	2.361	.018	
Insomnia	←‐	Consensu	0.032	0.059	0.546	.585	
Insomnia	←‐	Duratio	4.800	10.005	0.480	.631	
Insomnia	←‐	Comprehen_sibilit	0.022	0.097	0.229	.819	
PB23	←‐	Parental_Burnout	1.000				
PB22	←‐	Parental_Burnout	0.989	0.080	12.309	***	
PB21	←‐	Parental_Burnout	0.953	0.074	12.833	***	
PB20	←‐	Parental_Burnout	0.942	0.071	13.252	***	
PB19	←‐	Parental_Burnout	0.939	0.066	14.319	***	
PB18	←‐	Parental_Burnout	1.027	0.067	15.221	***	
PB17	←‐	Parental_Burnout	1.052	0.071	14.874	***	
PB16	←‐	Parental_Burnout	0.930	0.059	15.773	***	
PB15	←‐	Parental_Burnout	1.011	0.091	11.156	***	
PB14	←‐	Parental_Burnout	0.909	0.091	10.016	***	
PB13	←‐	Parental_Burnout	1.040	0.065	16.077	***	
PB12	←‐	Parental_Burnout	0.810	0.056	14.445	***	
PB11	←‐	Parental_Burnout	0.748	0.065	11.425	***	
PB10	←‐	Parental_Burnout	1.153	0.080	14.333	***	
PB9	←‐	Parental_Burnout	1.132	0.077	14.632	***	
PB8	←‐	Parental_Burnout	0.983	0.091	10.761	***	
PB7	←‐	Parental_Burnout	0.994	0.061	16.173	***	
PB6	←‐	Parental_Burnout	1.059	0.064	16.553	***	
PB5	←‐	Parental_Burnout	1.105	0.071	15.493	***	
PB4	←‐	Parental_Burnout	1.112	0.075	14.798	***	
PB3	←‐	Parental_Burnout	1.161	0.076	15.342	***	
PB2	←‐	Parental_Burnout	0.928	0.069	13.466	***	
PB1	←‐	Parental_Burnout	0.931	0.074	12.640	***	
S7	←‐	Hyper_somnia	1.000				
S3	←‐	Hyper_somnia	0.579	0.140	4.135	***	
S2	←‐	Hyper_somnia	0.663	0.207	3.204	.001	
S1	←‐	Hyper_somnia	1.102	0.220	5.009	***	
S10	←‐	Insomnia	1.000				
S9	←‐	Insomnia	1.856	0.300	6.189	***	
S8	←‐	Insomnia	1.119	0.208	5.391	***	
S6	←‐	Insomnia	0.579	0.157	3.691	***	
S5	←‐	Insomnia	1.327	0.239	5.545	***	
S4	←‐	Insomnia	0.580	0.164	3.531	***	
ES3	←‐	Comprehen_sibilit	1.000				
ES7	←‐	Comprehen_sibilit	0.615	0.199	3.092	.002	
ES9	←‐	Duratio	1.000				
ES19	←‐	Duratio	0.033	0.186	0.180	.857	
ES1	←‐	Consensu	1.000				
ES25	←‐	Consensu	0.161	0.177	0.911	.362	
ES16	←‐	Ruminatio	1.000				
ES22	←‐	Ruminatio	2.365	0.902	2.622	.009	

## References

[brb32688-bib-0001] Alimoradi, Z. , Broström, A. , Tsang, H. W. , Griffiths, M. D. , Haghayegh, S. , Ohayon, M. M. , Lin, C. Y. , & Pakpour, A. H. (2021). Sleep problems during COVID‐19 pandemic and its’ association to psychological distress: A systematic review and meta‐analysis. EClinicalMedicine, 36, 100916. 10.1016/j.eclinm.2021.100916 34131640 PMC8192091

[brb32688-bib-0002] Altena, E. , Baglioni, C. , Espie, C. A. , Ellis, J. , Gavriloff, D. , Holzinger, B. , Schlarb, A. , Frase, L. , Jernelöv, S. , & Riemann, D. (2020). Dealing with sleep problems during home confinement due to the COVID‐19 outbreak: Practical recommendations from a task force of the European CBT‐I Academy. Journal of Sleep Research, 29(4), e13052. 10.1111/jsr.13052 32246787

[brb32688-bib-0003] Altman, N. G. , Izci‐Balserak, B. , Schopfer, E. , Jackson, N. , Rattanaumpawan, P. , Gehrman, P. R. , Patel, N. P. , & Grandner, M. A. (2012). Sleep duration versus sleep insufficiency as predictors of cardiometabolic health outcomes. Sleep Medicine, 13(10), 1261–1270. 10.1016/j.sleep.2012.08.005 23141932 PMC3527631

[brb32688-bib-0004] Batmaz, S. , & Ozdel, K. (2015). Psychometric properties of the Turkish version of the Leahy Emotional Schema Scale‐II. Anatolian Journal of Psychiatry, 16, 23. 10.5455/apd.170597

[brb32688-bib-0005] Batmaz, S. , Ulusoy Kaymak, S. , Kocbiyik, S. , & Turkcapar, M. H. (2014). Metacognitions and emotional schemas: A new cognitive perspective for the distinction between unipolar and bipolar depression. Comprehensive Psychiatry, 55(7), 1546–1555. 10.1016/j.comppsych.2014.05.016 24974282

[brb32688-bib-0006] Calarco, J. M. , Anderson, E. , Meanwell, E. , & Knopf, A. (2020). “Let's not pretend it's fun”: How COVID‐19‐related school and childcare closures are damaging mothers’ well‐being. *SocArXiv*. 10.31235/osf.io/jyvk4

[brb32688-bib-0007] Cappuccio, F. P. , D'Elia, L. , Strazzullo, P. , & Miller, M. A. (2009). Quantity and quality of sleep and incidence of type 2 diabetes: A systematic review and meta‐analysis. Diabetes Care, 33(2), 414–420. 10.2337/dc09-1124 19910503 PMC2809295

[brb32688-bib-0008] Cluver, L. , Lachman, J. M. , Sherr, L. , Wessels, I. , Krug, E. , Rakotomalala, S. , Blight, S. , Hillis, S. , Bachman, G. , Green, O. , Butchart, A. , Tomlinson, M. , Ward, C. L. , Doubt, J. , & McDonald, K. (2020). Parenting in a time of COVID‐19. The Lancet, 395(10231), e64. 10.1016/s0140-6736(20)30736-4 PMC7146667 32220657

[brb32688-bib-0009] Clancy, F. , Prestwich, A. , Caperon, L. , Tsipa, A. , & O'Connor, D. B. (2020). The association between worry and rumination with sleep in non‐clinical populations: A systematic review and meta‐analysis. Health Psychology Review, 14(4), 427–448. 31910749 10.1080/17437199.2019.1700819

[brb32688-bib-0010] Curtis, R. G. , Olds, T. , Ferguson, T. , Fraysse, F. , Dumuid, D. , Esterman, A. , Hendrie, G. A. , Brown, W. J. , Lagiseti, R. , & Maher, C. A. (2021). Changes in diet, activity, weight, and wellbeing of parents during COVID‐19 lockdown. PLoS One, 16(3), e0248008. 10.1371/journal.pone.0248008 33657182 PMC7928513

[brb32688-bib-0011] Dellagiulia, A. , Lionetti, F. , Fasolo, M. , Verderame, C. , Sperati, A. , & Alessandri, G. (2020). Early impact of COVID‐19 lockdown on children's sleep: A 4‐week longitudinal study. Journal of Clinical Sleep Medicine, 16(9), 1639–1640. 32620188 10.5664/jcsm.8648 PMC7970607

[brb32688-bib-0012] Edwards, E. R. , Micek, A. , Mottarella, K. , & Wupperman, P. (2016). Emotion ideology mediates effects of risk factors on alexithymia development. Journal of Rational‐Emotive & Cognitive‐Behavior Therapy, 35(3), 254–277. 10.1007/s10942-016-0254-y

[brb32688-bib-0013] Edwards, E. R. (2019). Assessment of third wave therapy assumptions about the relation between emotional schemas and psychoemotional functioning . Doctoral dissertation, City University of New York.

[brb32688-bib-0014] Edwards, E. R. , & Wupperman, P. (2019). Research on emotional schemas: A review of findings and challenges. Clinical Psychologist, 23(1), 3–14.

[brb32688-bib-0015] Falavigna, A. , de Souza Bezerra, M. L. , Teles, A. R. , Kleber, F. D. , Velho, M. C. , Da Silva, R. C. , Mazzochin, T. , Santin, J. T. , Mosena, G. , de Braga, G. L. , Petry, F. L. , & de Lessa Medina, M. F. (2011). Consistency and reliability of the Brazilian Portuguese version of the Mini‐Sleep Questionnaire in undergraduate students. Sleep and Breathing, 15(3), 351–355. 20652835 10.1007/s11325-010-0392-x

[brb32688-bib-0016] Fornell, C. , & Larcker, D. F. (1981). Evaluating structural equation models with unobservable variables and measurement error. Journal of Marketing Research, 18(1), 39–50. 10.1177/002224378101800104

[brb32688-bib-0017] Gruber, J. , Eidelman, P. , & Harvey, A. G. (2008). Transdiagnostic emotion regulation processes in bipolar disorder and insomnia. Behaviour Research and Therapy, 46(9), 1096–1100. 10.1016/j.brat.2008.05.004 18684436

[brb32688-bib-0018] Guastella, A. J. , & Moulds, M. L. (2007). The impact of rumination on sleep quality following a stressful life event. Personality and Individual Differences, 42(6), 1151–1162. 10.1016/j.paid.2006.04.028

[brb32688-bib-0019] Harvey, A. (2002). A cognitive model of insomnia. Behaviour Research and Therapy, 40(8), 869–893. 10.1016/s0005-7967(01)00061-4 12186352

[brb32688-bib-0020] Harvey, A. G. , Stinson, K. , Whitaker, K. L. , Moskovitz, D. , & Virk, H. (2008). The subjective meaning of sleep quality: A comparison of individuals with and without insomnia. Sleep, 31(3), 383–393. 10.1093/sleep/31.3.383 18363315 PMC2276747

[brb32688-bib-0021] Hoevenaar‐Blom, M. P. , Spijkerman, A. M. , Kromhout, D. , van den Berg, J. F. , & Verschuren, W. M. (2011). Sleep duration and sleep quality in relation to 12‐year cardiovascular disease incidence: The MORGEN study. Sleep, 34(11), 1487–1492. 10.5665/sleep.1382 22043119 PMC3198203

[brb32688-bib-0022] Hosseini, S. M. , Seddighi, A. S. , Seddighi, A. , & Nikouei, A. (2020). Validity and reliability of the mini sleep questionnaire‐Persian version (MSQP). Journal of Sleep Disorders & Therapy, 9, 317. 10.35248/2167-0277.20.9.317

[brb32688-bib-0023] Jahrami, H. , BaHammam, A. S. , Bragazzi, N. L. , Saif, Z. , Faris, M. , & Vitiello, M. V. (2021). Sleep problems during the COVID‐19 pandemic by population: A systematic review and meta‐analysis. Journal of Clinical Sleep Medicine, 17(2), 299–313. 33108269 10.5664/jcsm.8930 PMC7853219

[brb32688-bib-0024] Kim, S. , Shin, Y. J. , Park, B. , Park, S. , & Shin, J. W. (2021). Advanced cognitive behavioral therapy for insomnia (CBT‐I) based on acceptance and commitment therapy compared with CBT‐I: A pilot study. Journal of Sleep Medicine, 18(2), 78–87. 10.13078/jsm.210002

[brb32688-bib-0025] Kripke, D. F. , Garfinkel, L. , Wingard, D. L. , Klauber, M. R. , & Marler, M. R. (2002). Mortality associated with sleep duration and insomnia. Archives of General Psychiatry, 59(2), 131. 10.1001/archpsyc.59.2.131 11825133

[brb32688-bib-0026] Leahy, R. L. (2015). Emotional schema therapy. Guilford Publications.

[brb32688-bib-0027] Leahy, R. L. (2019). Emotional schema therapy. New York: Guilford Publications.

[brb32688-bib-0028] Leahy, R. L. , Tirch, D. D. , & Melwani, P. S. (2012). Processes underlying depression: Risk Aversion, emotional schemas, and psychological flexibility. International Journal of Cognitive Therapy, 5(4), 362–379. 10.1521/ijct.2012.5.4.362

[brb32688-bib-0029] Lin, Y. N. , Liu, Z. R. , Li, S. Q. , Li, C. X. , Zhang, L. , Li, N. , Sun, X. W. , Li, H. P. , Zhou, J. P. , & Li, Q. Y. (2021). Burden of sleep disturbance during COVID‐19 pandemic: A systematic review. Nature and Science of Sleep, 13, 933–966. 10.2147/nss.s312037 PMC8253893 34234598

[brb32688-bib-0030] Medic, G. , Wille, M. , & Hemels, M. E. (2017). Short‐ and long‐term health consequences of sleep disruption. Nature and Science of Sleep, 9, 151–161. 10.2147/NSS.S134864 PMC5449130 28579842

[brb32688-bib-0031] Mikolajczak, M. , Brianda, M. E. , Avalosse, H. , & Roskam, I. (2018). Consequences of parental burnout: Its specific effect on child neglect and violence. Child Abuse & Neglect, 80, 134–145. 29604504 10.1016/j.chiabu.2018.03.025

[brb32688-bib-0032] Mousavi, S. F. (2020). Psychological well‐being, marital satisfaction, and parental burnout in Iranian parents: The effect of home quarantine during COVID‐19 outbreaks. Frontiers in Psychology, 11, 553880. 10.3389/fpsyg.2020.553880 33343439 PMC7744775

[brb32688-bib-0033] Mousavi, S. F. , Mikolajczak, M. , & Roskam, I. (2020). Parental burnout in Iran: Psychometric properties of the Persian (Farsi) version of the Parental Burnout Assessment (PBA). New Directions for Child and Adolescent Development, *174*, 85–100. 10.1002/cad.20369 33029877

[brb32688-bib-0034] Nomaguchi, K. , & Milkie, M. A. (2020). Parenthood and well‐being: A decade in review. Journal of Marriage and Family, 82(1), 198–223. 32606480 10.1111/jomf.12646 PMC7326370

[brb32688-bib-0035] Palagini, L. , Ong, J. C. , & Riemann, D. (2017). The mediating role of sleep‐related metacognitive processes in trait and pre‐sleep state hyperarousal in insomnia disorder. Journal of Psychosomatic Research, 99, 59–65. 10.1016/j.jpsychores.2017.03.001 28712431

[brb32688-bib-0036] Palagini, L. , Piarulli, A. , Lai, E. , Cheli, E. , Espie, C. , & Gemignani, A. (2013). Metacognition selectively defines primary insomnia. Sleep Medicine, 14, e228. 10.1016/j.sleep.2013.11.549 24916095

[brb32688-bib-0037] Petts, R. J. , Carlson, D. L. , & Pepin, J. R. (2021). A gendered pandemic: Childcare, homeschooling, and parents' employment during COVID‐19. Gender, Work & Organization, 28, 515–534.

[brb32688-bib-0038] Perini, F. , Wong, K. F. , Lin, J. , Hassirim, Z. , Ong, J. L. , Lo, J. , Ong, J. C. , Doshi, K. , & Lim, J. (2021). Mindfulness‐based therapy for insomnia for older adults with sleep difficulties: A randomized clinical trial. Psychological Medicine, 1–11. Online ahead of print. 10.1017/s0033291721002476 PMC9975962 34193328

[brb32688-bib-0039] Pillai, V. , & Drake, C. L. (2015). Sleep and repetitive thought: The role of rumination and worry in sleep disturbance. K. Babson , & M. Feldner (Eds.), Sleep and Affect: Assesment, Theory and Clinical Implications, (pp. 201–225). Academic Press.

[brb32688-bib-0040] Roskam, I. , & Mikolajczak, M. (2020). Gender differences in the nature, antecedents and consequences of parental burnout. Sex Roles: A Journal of Research, 83(7–8), 485–498. 10.1007/s11199-020-01121-5

[brb32688-bib-0041] Roskam, I. , Brianda, M. E. , & Mikolajczak, M. (2018). A step forward in the conceptualization and measurement of parental burnout: The Parental Burnout Assessment (PBA). Frontiers in Psychology, 9, 758. 10.3389/fpsyg.2018.00758 29928239 PMC5998056

[brb32688-bib-0042] Salari, N. , Khazaie, H. , Hosseinian‐Far, A. , Khaledi‐Paveh, B. , Ghasemi, H. , Mohammadi, M. , & Shohaimi, S. (2020). The effect of acceptance and commitment therapy on insomnia and sleep quality: A systematic review. BMC Neurology, 20(1), 10.1186/s12883-020-01883-1 32791960 PMC7425538

[brb32688-bib-0043] Salemi‐Langroudi, A. , Dobson, K. S. , Artounian, V. , Ghasemi, M. , Kolahkaj, B. , Khosravani, V. , Shafaghi, M. , Bafekr, T. , Heidarian, A. , Behfar, Z. , & Kiani Dehkordi, M. (2021). Psychometric properties of the leahy emotional schema scale‐II among Iranian students. International Journal of Cognitive Therapy, 14(3), 455–472. 10.1007/s41811-021-00111-z

[brb32688-bib-0044] Schmidt, A. , Kramer, A. C. , Brose, A. , Schmiedek, F. , & Neubauer, A. B. (2021). Distance learning, parent–child interactions, and affective well‐being of parents and children during the COVID‐19 pandemic: A daily diary study. Developmental Psychology, 57(10), 1719–1734. 10.1037/dev0001232 34807692

[brb32688-bib-0045] Sella, E. , Cellini, N. , Miola, L. , Sarlo, M. , & Borella, E. (2018). The influence of metacognitive beliefs on sleeping difficulties in older adults. Applied Psychology: Health and Well‐Being, 11(1), 20–41. 10.1111/aphw.12140 30338645

[brb32688-bib-0046] Silberstein, L. R. , Tirch, D. , Leahy, R. L. , & McGinn, L. (2012). Mindfulness, psychological flexibility and emotional schemas. International Journal of Cognitive Therapy, 5(4), 406–419.

[brb32688-bib-0047] Sinha, M. , Pande, B. , & Sinha, R. (2020). Impact of COVID‐19 lockdown on sleep‐wake schedule and associated lifestyle related behavior: A national survey. Journal of Public Health Research, 9(3), . 10.4081/jphr.2020.1826 PMC7445442 32874967

[brb32688-bib-0048] Sher, L. (2020). COVID‐19, anxiety, sleep disturbances and suicide. Sleep Medicine, 70, 124–124. 32408252 10.1016/j.sleep.2020.04.019 PMC7195057

[brb32688-bib-0049] Suh, J. W. , Lee, H. J. , Yoo, N. , Min, H. , Seo, D. G. , & Choi, K. H. (2019). A brief version of the Leahy Emotional Schema Scale: A validation study. International Journal of Cognitive Therapy, 12(1), 38–54.

[brb32688-bib-0050] Taylor, H. L. , Hailes, H. P. , & Ong, J. (2015). Third‐wave therapies for insomnia. Current Sleep Medicine Reports, 1(3), 166–176. 10.1007/s40675-015-0020-1

[brb32688-bib-0051] Thorell, L. B. , Skoglund, C. , de la Peña, A. G. , Baeyens, D. , Fuermaier, A. B. , Groom, M. J. , Mammarella, I. C. , van der Oord, S. , van den Hoofdakker, B. J. , Luman, M. , de Miranda, D. M. , Siu, A. F. Y. , Steinmayr, R. , Idrees, I. , Soares, L. S. , Sörlin, M. , Luque, J. L. , Moscardino, U. M. , Roch, M. , & Christiansen, H. (2021). Parental experiences of homeschooling during the COVID‐19 pandemic: Differences between seven European countries and between children with and without mental health conditions. European Child & Adolescent Psychiatry, 31, 649–661. 33415470 10.1007/s00787-020-01706-1 PMC7790054

[brb32688-bib-0052] Tsypes, A. , Aldao, A. , & Mennin, D. S. (2013). Emotion dysregulation and sleep difficulties in generalized anxiety disorder. Journal of Anxiety Disorders, 27(2), 197–203. 10.1016/j.janxdis.2013.01.008 23474909

[brb32688-bib-0053] Stuck, B. A. , Maurer, J. T. , Schlarb, A. A. , Schredl, M. , & Weeß, H. (2021). Practice of sleep medicine: Sleep disorders in children and adults. Springer.

[brb32688-bib-0054] Vandekerckhove, M. , & Wang, Y. L. (2017). Emotion, emotion regulation and sleep: An intimate relationship. AIMS Neuroscience, 5(1), 1–17. 10.3934/Neuroscience.2018.1.1 32341948 PMC7181893

[brb32688-bib-0055] Wearick‐Silva, L. E. , Richter, S. A. , Viola, T. W. , & Nunes, M. L. (2021). Sleep quality among parents and their children during COVID‐19 pandemic. Jornal de Pediatria, 98, 248–255. 10.1016/j.jped.2021.07.002 PMC8432904 34480854

[brb32688-bib-0056] Zomer, J. , Peled, A.‐H. , Rubin, E. , & Lavie, P. (1985) Mini‐sleep Questionnaire (MSQ) for screening large populations for EDS complaints. In: W. P. Koella , E. Rüther , H. Schulz (Eds.). Sleep '84: Proceedings of the Seventh European Congress on Sleep Research. Stuttgart: Fischer, pp. 467–470.

